# Supplementary material for: Disease-linked regulatory DNA variants and homeostatic transcription factors in epidermis
Source: Nat Commun. 2025 Sep 25;16:8387. doi: 10.1038/s41467-025-63070-5 (PMC12462481; doi:10.1038/s41467-025-63070-5)
Supplement: Supplementary file 1 — Supplementary Information [file 41467_2025_63070_MOESM1_ESM.pdf]

Supplementary Discussion

Identification of rs4687102 as a dynamic element during differentiation

The disease-linked rs4687102 SNV breaks an ATF1/2/4 motif near the TP63 gene (**Supplementary Fig 5a-b**). rs4687102 risk and protective alleles were differentially active in driving transcription in MPRA (**Supplementary Fig 5c**). To examine potential ATF4 binding to rs4687102, CUT&RUN of ATF4 in a heterozygous individual was performed, revealing that the risk allele bound ATF4 at 68% of the level as the protective allele (**Supplementary Fig. 5d**, *inset*, P=0.06, two-sided binomial). Other studies in other tissues have found CEBPG, CEBPB, and ATF2/3/4 bound at this SNV, which could also be affected TFs[1]. These data propose a potential model in which rs4687102 SNV-mediated differences in the binding of ATF4 (or potentially CEBPB/G) to an enhancer dysregulates *TP63* expression in a manner that may predispose to the emergence of the altered epidermal homeostasis observed in lesional psoriatic tissue and suggest a rationale for additional studies of disease-linked SNV impacts on TF DNA binding.

Supplementary Figure Legends

Supplementary Fig. 1. Sankey diagram of polygenic skin disease incidence. Incidence numbers reflect rates among U.S. persons, all ages, both sexes, 2021 GDB data[2] (with a few exceptions, see Methods). A rate of 1 per 100 person-years corresponds to one diagnosis per 100 people per year.

Supplementary Fig. 2. Flow chart of the high throughput experiments in this work. Blue boxes represent experiments, and green boxes represent analytical results. CUT&RUN candidates were determined from the union set of motifs enriched in MPRA, CRISPR screen hits, and TFs established in skin in the literature.

Supplementary Fig. 3. Correlations in disease risk genes for polygenic skin disease. **a** LDSC genetic correlation of 9 skin disease GWAS. Genetic correlations that surpassed significance thresholds following Benjamini–Hochberg (FDR) correction are labeled as follows: FDR < 0.05 ~ *, 0.01 ~ **, 0.001 ~ ***, 0.0001 ~ ****. **b** Overlap between diseases among the alleles used for MPRA. Inflammatory skin disease is marked with an asterisk because it included other inflammatory diseases listed separately, explaining the high overlap with psoriasis. **c** LDSC heritability enrichment of primary epithelial cell and GM12878 ATAC peaks for 9 skin disease studies. Enrichments surpassing both unadjusted (yellow) and FDR-adjusted (red) p-value thresholds (<0.1) are highlighted. **d** LDSC heritability enrichment of immune cell ATAC peaks for 2 atopic dermatitis studies.

Supplementary Fig. 4. MPRA quality metrics and overview. a, The location of skin disease SNVs in human chromosomes highlights the epidermal differentiation complex and the HLA locus. **b**, Number of variants by disease. **c**,The number of unique reads per MPRA dataset shows no large difference between samples. **d**, The count frequency per barcode for 82,555 barcodes is not overly skewed. μ, mean count frequency. **e**, Nearly all fragments had all 10 barcodes observed in the plasmid DNA of the MPRA library. **f**, Nearly all fragments had 9-10 out of 10 barcodes observed in the final RNA library. A similarly high recovery was observed for the CRISPR knock-out MPRA libraries. **g,** Day 3 and 6 correlate better in MPRA data than

with Day 0. **h**, Replicate correlation in MPRA was robust. **i**, The number of active fragments in each condition relative to random controls does not vary greatly. "Safe" denotes cells treated with a safe targeting control guide, while SNAI2/IRF6/NRF1 denote cells treated with guides for the indicated TF. **j**, Enrichment of fragment activity by chromVAR category shows that elements from active TSS are enriched for increased transcriptional activity (color: 2-sided t-test P value). **k**, Enrichment of changes in transcriptional activity across the differentiation time-course by chromVAR category identifies TSS elements as more likely to be dynamic across differentiation, and to be most active at Day 3. **l**, Sample correlation in CRISPR KO-MPRA libraries show IRF6 knock-out Day 3 cells have an undifferentiated phenotype. "Safe" denotes cells treated with a safe targeting control guide.

Supplementary Fig. 5. rs4687102 is a differentially active allele linked to TP63. a, rs4687102 alters an ATF motif. **b,** rs4687102 resides in an enhancer with increased H3K27ac and looping during differentiation, and is bound by ATF4. The SNV is H3K27ac HiChIP-looped to the promoter of the TP63 gene. **c**, rs4687102 is differentially active in MPRA. **d**, ATF4 binds to the risk allele in CUT&RUN data (*insert*). ASB, allele specific binding. RPM, reads per million.

Supplementary Fig. 6. SNVs in MPRA that differ in activity by allele. a, Enrichment of differential risk/protective activity by chromVAR category suggests a depletion of differential activity from "strong transcription" regions. **b**, Top outlier daSNVs with the most extreme allelic effects. **c**, Motif activity by JASPAR motif category. "g" denotes Hedge's g (effect size). P values, two-sided Mann-Whitney for the motif being associated with a change in transcriptional activity. **d**, The atopic dermatitis daSNV rs72696969 is connected via looping to the promoters of FLG and FLG2. **e**, The risk "A" allele of rs72696969 has lower transcriptional activity in MPRA. **f**, HDR edits of primary keratinocytes results in reduced FLG expression for the risk-associated rs72696969 "A" allele. PA2G4 and ERBB3 are nearby control genes. The panel on the right is Sanger sequencing traces of the input and edited populations. Expression was evaluated in progenitor cells. Whiskers, 95% CI. **g**, The rs2349075 and rs2540334 loci, which are eQTLs for CASP8 and are associated with skin cancer. Dotted lines indicate the position of indicated variants. **h**, IRF6 has reduced binding to the risk allele of rs2540334 by microscale thermophoresis. **i,** Differential activity in MPRA of rs2540334 shows reduced activity for the risk variant. **j**, The risk allele of rs2349075 has decreased transcriptional activity in MPRA. **k**, CUT&RUN of JUN and JUNB show decreased binding to the risk allele of rs2349075 in a heterozygous individual. ASB, allele-specific binding event. **l**, JUN, but not the control TF YY1, shows decreased binding to the risk allele of rs2349075 when expressed from the PROBER vector and assayed by proximity biotinylation[3]. In PROBER, a promiscuous biotin ligase "sprayer" is tethered to a vector bearing the protective or risk alleles, then biotinylated proteins are purified and assayed by immunoblot. **m**, Edits to the endogenous rs2349075 locus in primary keratinocytes from a heterozygous individual shows decreased CASP8 expression for the risk allele. CFLAR is a nearby control gene. The panel on the right is Sanger sequencing traces of the input and edited populations. Whiskers, 95% CI.

Supplementary Fig. 7. CRISPR-flow. a, The correlation of SSC-A and KRT10 staining in CRISPR-flow agrees with prior results that cells enlarge and become more granular across differentiation. **b**, The number of UMIs by sample in CRISPR-flow. **c,** Essential gene guides are depleted from cells. UMIs per million UMIs were summed across all samples, then the plasmid DNA library ("pDNA") UMI-per-million count is subtracted to normalize to the input

distribution. Essential genes are as defined by DepMap. **d**, Guides targeting DepMap "selective" genes (e.g., genes that reduce growth) and keratinocyte-specific factors were depleted by growth and a subset of TFs were enriched by growth. **e**, Combining high/low enrichment statistics into a single pseudo-P value for an effect leads to a depletion of un-expressed genes (see methods). P values for enrichment or depletion from the KRT10 high or low samples were combined into a pseudo-P value and the effect of different cutoffs (top panel) was evaluated for the depletion of un-expressed genes (middle panel, fraction of the expected number of unexpressed genes included, bottom panel, significance of the depletion of unexpressed genes by Fisher's exact test). **f**, Calculation of an FDR rate for pseudo-P cutoffs based on the depletion of un-expressed genes as a function of prior belief in the true positive (TP) number. **g**, Diagram of how effects on growth can result in effects on the distribution of guides between progenitor and differentiated cells. **h**, The number of UMIs per gene as a histogram for genes in the given categories. **i**, PCA of guide enrichments reveal a primary axis of promoting/repressing differentiation, and a secondary axis correlated with whether the TF is more essential under differentiation or progenitor growth conditions.

Supplementary Fig. 8. Overview of perturb-seq experiments. **a**, Keratinocytes were seeded for differentiation and harvested at the indicated timepoint for perturb-seq. "# usable cells" is the cells used for statistical analysis of effects on the pseudotime distribution (see Methods). **b**, RNA velocity analysis of Day 6 differentiated keratinocytes shows an RNA-velocity derived trajectory matching the pseudotime curve. This UMAP differs due to it being calculated by a different program (scanpy) based on a smaller subset of cells. **c**, Transcription factors are successfully depleted in target cells. The pink distribution represents the distribution of Z scores for expression of the target TF RNA in cells with guides against the target TF. Only TFs in the top half of expression are included, due to the limited sensitivity of single cell RNA-seq. **d**-**e**, Guide effects on differentiation in perturb-seq as determined by Mann-Whitney tests for altered pseudotime trajectory at day 3 and day 6. **f**, Correlation of differentiation effects between split-pool and 10X-based sequencing.

Supplementary Fig. 9. Gene set enrichments in homeostasis TFs. **a**, Enrichment of the 123 homeostasis TFs for TF PPIs, followed by transcription factor networks **b** and co-expression **c**.

Supplementary Fig. 10. Perturb-seq and inflammation. **a**, Expression of three keratinocyte inflammation factors in Day 3 UMAPs in 10x data. **b**, Expression of pro-inflammatory IL1 family cytokines IL1A, IL1B and IL18 in Day 3 UMAPs. **c**, Expression of IL1 cytokines across differentiation either in cells with any guide (solid lines) or with ATF4 or HIF1A knock-outs (dashed lines). **d**, RNA velocity and expression analysis of basal marker ITGA6, differentiation marker KRTDAP, IL1 cytokines (IL1A/B, IL18) and the IL1 receptor IL1R2 in Day 6 differentiated keratinocytes. A positive (green) velocity value represents induction, and negative (red) repression. Pseudotime increases left-to-right. **e**, Correlation of IL1 cytokine regulation and differentiation effects for TF knock-outs reveals only a slight negative correlation after regressing out pseudotime effects. **f**, Inflammation effects for TFs controlling for effects on differentiation. **g**, TFs with significant effects on inflammation, FDR<0.2. **h**, ATF4 and TFAP2A bind proximal to IL1A/B. ATF4 binding is reduced 2-fold over differentiation, consistent with a loss of activation at the later time points.

Supplementary Fig. 11 Novel TF validation. a, Target TFs were successfully depleted by siRNAs, as determined by DESeq2 from bulk RNA-seq. **b**, GO analysis of differential genes in bulk RNA-seq, compared to expressed genes. **c**, Number of CUT&RUN peaks FDR<0.01. **d**, CUT&RUN enriched motifs of the novel epidermal TFs and SP1 match known motifs for factors with established motifs (NRF1, SP1), or general sequence preference (CXXC1). The right inset shows the fraction of peaks with the given motif ("Peaks") or the fraction of length-matched random sequence controls ("Random") with the given motif, as reported by DREME. **e**-**f**, Changes in motif location categories across differentiation and their significance (t-test, 2-sided). **g**, Binding at the YWHAZ locus. **h**, TSS peaks and RNA expression changes on siRNA knock-down were correlated for CXXC1 and SP1. The increased significance for the overlap of induced genes with binding suggests an activating effect on balance.

Supplementary Fig. 12 Homeostasis TF binding. a, Number of FDR<0.05 MACS2 peaks per condition. **b**, Number of CUT&RUN samples per condition. **c**, Mean linear correlation coefficients between replicates for CUT&RUN samples, based on the maximum read depth observed in a random subset of the union peak set. **d**-**e**, PCA plot of peak heights in CUT&RUN place similar proteins together. The union peak set is determined by a random 200 peak subset of the top 500 peaks for each condition, then those peaks merged across all conditions.

Supplementary Fig 13 Co-binding analysis. **a**, fraction of peaks that overlap between the indicated TFs. The overlap is counted as a fraction of the top 2,000 peaks for the given column. Peaks were downsampled to the number of peaks in the smallest peak set (916) so that the denominator was the same for (A & B)/A and (A & B)/B. All except two datasets had >2,000 peaks. **b**, Absolute (non-differential) enrichment of TF motifs in CUT&RUN peaks by Centrimo. Adjusted p values as calculated by Centrimo with default parameters. **c,** Differential (Day 4 vs Day 0) Centrimo analysis[4] of TF motif enrichment in CUT&RUN peaks. Differential enrichment was run for each direction of change (Day 4 vs Day 0 and Day 0 vs Day 4), and the largest absolute value of the log Fisher adjusted p-value for differential enrichment was plotted. If the motif was more enriched in progenitors, the log p value was made negative.

Supplementary Fig 14 Homeostasis TF peak analysis. a, MACS2 peak significance for specific keratinocyte differentiation genes and progenitor markers for the indicated TFs. **b**, Enrichment in peak locations *vs* random expressed genes for skin homeostasis genes (skin genes defined by GO). **c**, Enrichment of peaks in skin genes (*left*) and monogenic skin disease genes (*right*), for the indicated region. "Proteins separate" is the average across TFs (P value, two-sided t-test). Proteins combined is the combination of peaks across all TFs, followed by empirical P value determination (one-sided). Whiskers, 95% CI. **d**, As panel **b**, but for monogenic skin disease genes.

Supplementary Fig 15 Promoters are buffered against allele-specific binding. a Mean |log2| in reads at ASBs as a function of genomic location. P values, two-sided t-tests. Box plot, 25-75% data range; center line, median; whiskers,1.5 IQR; outliers not plotted. Overlay, mean and 95% CI. **b** A lack of read depth dependence suggests background reads do not create the buffering effect. Left axis and green dots, ratio of |log2(allele1/allele2)| values (buffering); right axis and black/red dots, mean read numbers in the indicated bin. **c**, ANOVA coefficients for SNV location, regressing |log2(allele1/allele2)| values on TF, SNV location and read depth. P

values, two-sided t-tests for coefficients ≠ zero. **d**, Kernel density estimate plot of the population frequency of an allele *vs* the binding ratio (in log2 reads) of the second allele (allele 1 is picked randomly between reference/alternate). Binding correlates with allele frequency with very low correlation (R=0.05). Red lines, ±log2(0.3); the region inside the red lines is set to zero for easier visualization. **e** Motif clustering at bound heterozygous sites. Motifs were considered in a cluster if their start positions were 2-50 nucleotides apart. P values for this panel and the following are two-sided Mann-Whitney U tests. This is a boxenplot, depicting the median as center line, 50% of the data in the center two boxes, and each successive set of thinner boxes containing half of the remaining data; values clipped to 10, log2(N) – 3 box levels drawn. Overlay, mean and 95% CI. P values for this panel and the following are two-sided Mann-Whitney U tests. **f** Information content at SNV modified bases. P values were P>0.1 except TFAP2A. Overlay, mean and 95% CI. Box plot parameters are as in panel (a). **g** Fraction of the 141 bp region around bound heterozygous SNVs that are covered by a cognate motif. Box plot, 25-75% data range; center line, median; whiskers, 10-90% data range. Overlay, mean and 95% CI. **h** Fraction of bound heterozygous sites with the SNV in a cognate motif. Whiskers, 95% CI. **i** Fraction of bound heterozygous sites with a modified cognate motif. Whiskers, 95% CI.

Supplementary Fig. 16 ASBs and TF occupancy. **a** "HOT" regions have reduced ASB fold changes. "HOT" regions denote regions sensitive to DNAse I in epithelial keratinocytes (generally ~10 kbp) with a high number of bound TFs, as defined in by the "wgEncodeUWDukeDnaseNHEK" dataset. Non-HOT regions were DNAse I sensitive but without the high number of bound TFs. "Non-DNAse regions" were regions not listed in the NHEK DNAse sensitivity dataset. Peaks were subset to those with at least 50 reads over the SNV. P values, two-sided Mann-Whitney U. This is a boxenplot, which depicts the median as the center line, and 50% of the data in the center two boxes, with each successive set of thinner boxes containing half of the remaining data. The overlay shows the mean and 95% CI. Box levels were log2(N) – 3 for N datapoints. **b** ASB fold changes in promoter SNVs for TFs with at least 50 bound heterozygous sites in promoters correlated with the number of TFs in this dataset bound to the same promoter. That is, the greater the number of different TFs at the promoter, the more equal the number of reads mapping to each allele in a SNV. P values, Spearman rank correlation.

Supplementary Fig. 17 ASB enrichment near homeostasis genes. a, Putative ASBs are enriched near skin homeostasis genes and monogenic epithelial disease genes. "Proteins separate" is the average across TFs (P value, two-sided t-test, for the average TF's ASBs being unrelated to the indicated gene group). Proteins combined is the combination of ASBs across all TFs, followed by comparing overlap *vs* random expressed gene selection and empirical P value determination (one-sided). Whiskers, 95% CI. **b**, Putative ASBs are enriched near monogenic epithelial disease genes. P value is determined from comparing overlap *vs* random gene selection.

Supplementary Fig. 18 ASB features a, For skin disease associated SNVs, there is a positive correlation in SP/KLF TF CUT&RUN reads for risk vs protective alleles with transcriptional activity from the same alleles. **b**, Upset plot of FDR < 0.01 daSNV "validation" through eQTL (GTEx), or binding/ASB by a homeostasis TF (this work). Three SNVs were also subject to genomic edits. **c**, Sanger sequencing traces of the rs4704864 input and edited populations.

Supplementary Methods

Reagents

Reagent	Company	Cat. #
**Antibodies**		
anti-HA	CST	#3724
anti-H3K27me3	Active Motif	#39055
anti-V5	CST	#13202
anti-ATF4	ProteinTech	10835-1-AP
anti-SP1	ProteinTech	21962-1-AP
anti-CXXC1	ProteinTech	27963-1-AP
anti-SP3	ProteinTech	26584-1-AP
anti-KLF4	Sigma	HPA002926
anti-KLF4	Bio-Techne	AF3640
anti-c-JUN	Abcam	ab32137
anti-CREB1	Abcam	ab32096
anti-YY1	Abcam	ab109228
anti-SNAI2	CST	#9585
anti-TFAP2A	SCBT	sc-12726/25343
anti-AHR	SCBT	sc-133088
anti-TEAD1	Abcam	ab133533
anti-JUNB	SCBT	sc-8051
anti-OVOL1	ProteinTech	14082-1-AP
anti-TADA2B	ProteinTech	67439-1-Ig
anti-SOX9	ProteinTech	17367-1-AP
anti-GRHL1	ProteinTech	17644-1-AP
anti-P63	CST	#4892
anti-KLF5	ProteinTech	21017-1-AP
anti-RUNX1	ProteinTech	25315-1-AP
anti-TEAD3	CST	13224
anti-DLX3	ProteinTech	13261-3-AP

Reagent	Company	Cat. #
anti-ETV3	Aviva	ARP85957
anti-KHDRBS1	Proteintech	10222-1-AP
anti-HOPX	ProteinTech	11419-1-AP
IgG	CST	#2729
IgG	SCBT	sc-2025
anti-KRT10	Novus Bio	NBP2-47825AF647
**Cell Lines**		
Lenti-X 293T	Takara Bio	#632180
Human primary normal skin cells	Stanford University School of Medicine	GDS
**Reagents and Materials**		
anti-FLAG M2 affinity gel	Millipore	A2220
Amicon 3k cutoff columns	Millipore	UFC500396
Stellar Competent Cells	Takara Bio	#636763
DMEM	Gibco	#11995-065
Keratinocyte-SFM	Gibco	#17005-042
Medium 154	Gibco	#M-154-500
rEGF	Gibco	#10450-013
BPE	Gibco	13028-014
Pen/Strep	Life Technologies	15140-122
Lipofectamine 3000	Invitrogen	L3000015
Lipofectamine RNAiMax	Invitrogen	13778150
Lenti-X concentrator	Takara Bio	631231
Monolith His-Tag Labeling Kit	NanoTemper	MO-L018
Monolith NT.115 Capillaries	NanoTemper	MO-K022
RNeasy Plus Kit	QIAGEN	74136
Dynabeads mRNA DIRECT purification kit	ThermoFisher	61011
SuperScript IV	ThermoFisher	18090050
AMPure XP beads	Beckman Coulter	A63880
SYBR green	ThermoFisher	S7563

Reagent	Company	Cat. #
PrimeStar Max DNA Polymerase	Takara	R045B
Cell Staining Buffer	Biolegend	#420201
Fix/Perm buffer	Biolegend	#426803
Perm/Wash buffer	Biolegend	#426803
RNAse A	Qiagen	#19101
Proteinase K	ThermoFisher	AM2546
InstantBlue Protein Stain	Abcam	ab119211
Zymo PCR cleanup kit	Zymo	D4013/#D4029
QuantSeq 3' mRNA-Seq V2 Library Prep Kit FWD with UDI	Lexogen	191.96
UMI Second Strand Synthesis Module for QuantSeq FWD	Lexogen	018.96
Protein G dynabeads	Invitrogen	10004D
anti-HA magnetic beads	ThermoFisher	#88837
CUTANA™ ChIC/CUT&RUN Kit	EpiCypher	14-1048
Concanavalin A beads	EpiCypher	21-1401
pAG-MNase	EpiCypher	15-1016
Zymo DNA Clean and Concentrator	Zymo Research	D4014
NEBNext Ultra II DNA library prep kit	NEB	E7103S
**Plasmids**		
pLEX-FHH-Empty Vector-IRES-Puro	Addgene	#120568
pLEX-FHH-ZNF217	Addgene	#25648
pLEX-FHH-NRF1	Addgene	#212207

References

1. Boyle, A. P. *et al.* Annotation of functional variation in personal genomes using RegulomeDB. *Genome Res* **22**, 1790–1797 (2012).

2. Global Burden of Disease Collaborative Network. Global Burden of Disease Study 2021 (GBD 2021). *Institute for Health Metrics and Evaluation (IHME)* (2024).

3. Mondal, S. *et al.* PROBER identifies proteins associated with programmable sequence-specific DNA in living cells. *Nat Methods* **19**, 959–968 (2022).

4. Lesluyes, T., Johnson, J., Machanick, P. & Bailey, T. L. Differential motif enrichment analysis of paired ChIP-seq experiments. *BMC Genomics* **15**, 752 (2014).

Figure S1

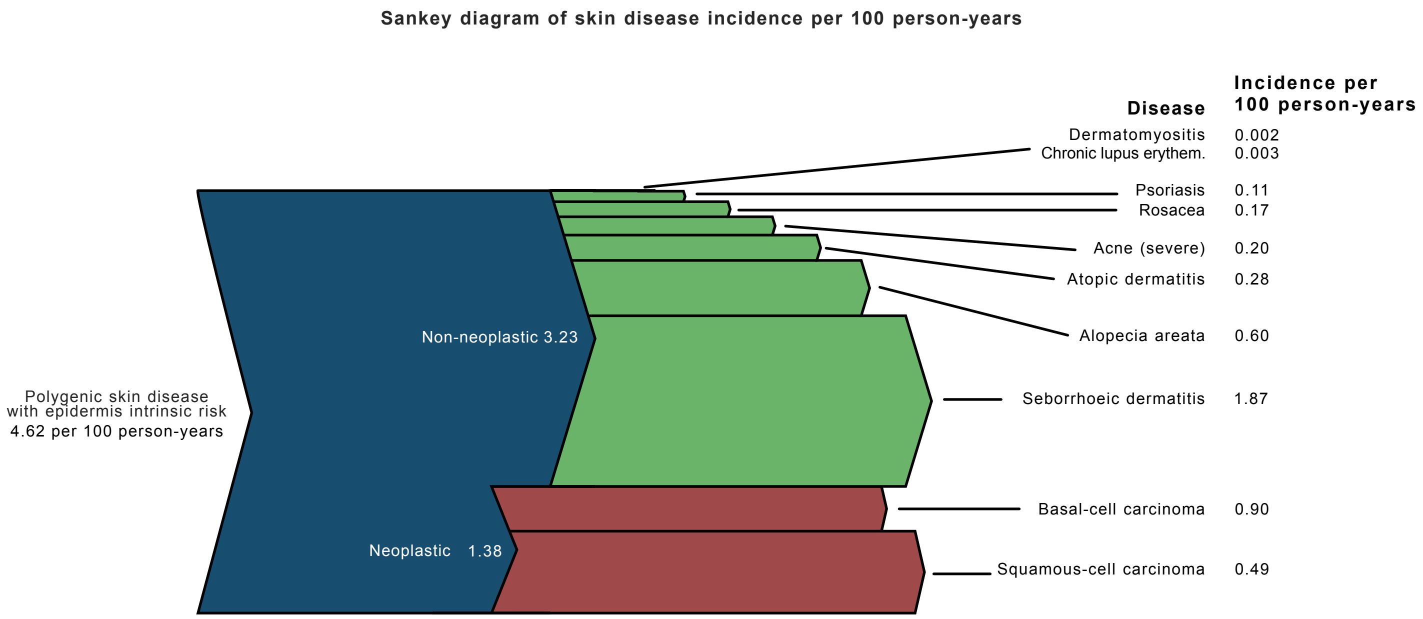

Sankey diagram of skin disease incidence per 100 person-years
Disease
Incidence per 100 person-years
Dermatomyositis 0.002
Chronic lupus erythem. 0.003
Psoriasis 0.11
Rosacea 0.17
Acne (severe) 0.20
Atopic dermatitis 0.28
Alopecia areata 0.60
Seborrhoeic dermatitis 1.87
Basal-cell carcinoma 0.90
Squamous-cell carcinoma 0.49
Non-neoplastic 3.23
Neoplastic 1.38
Polygenic skin disease
with epidermis intrinsic risk
4.62 per 100 person-years

Figure S2

Overview of high-throughput experiments

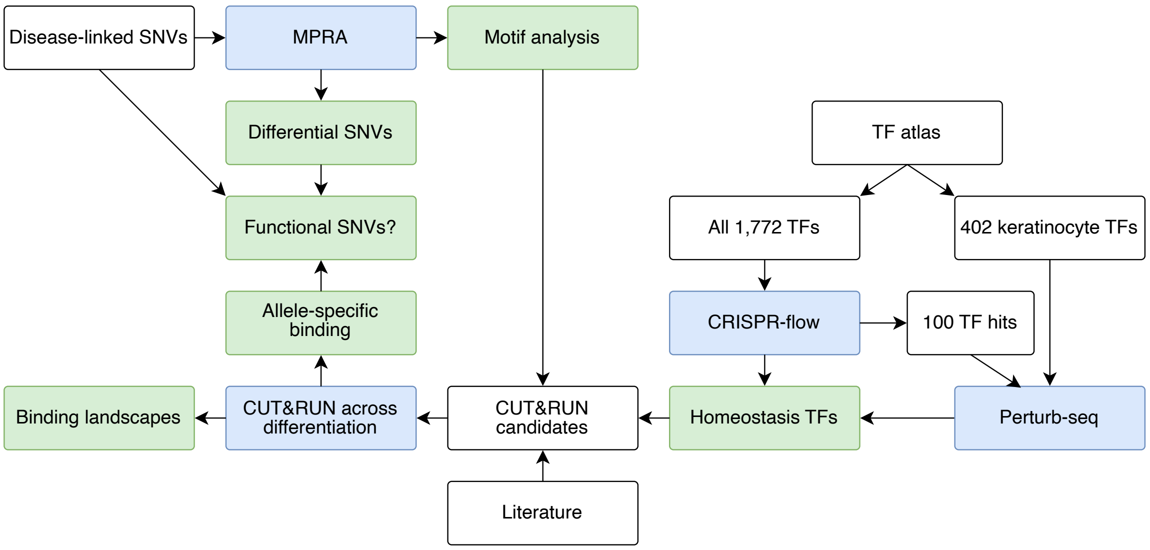

Figure S3

LD analysis

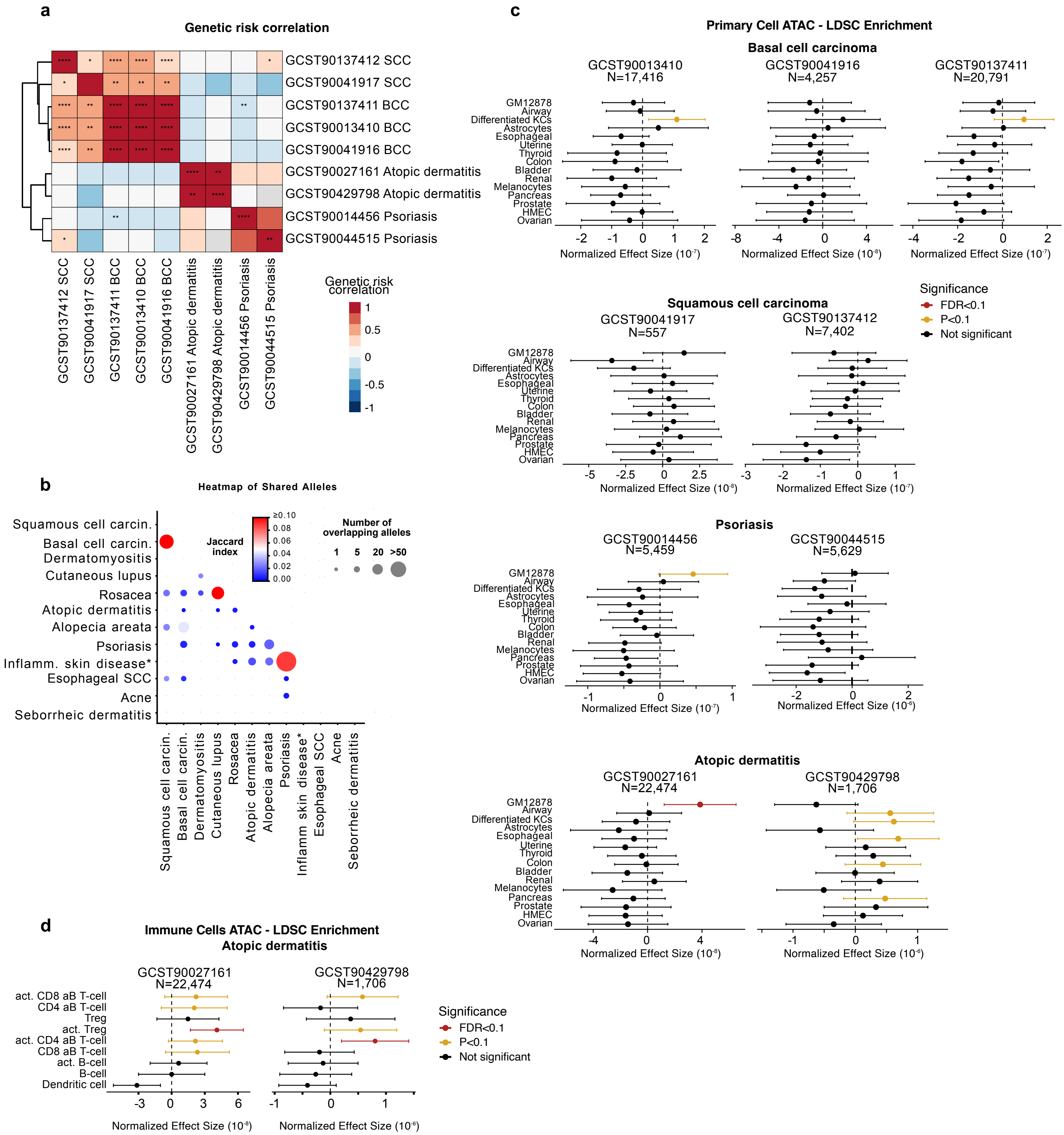

Figure S4

Skin disease linked SNVs MPRA overview and data quality

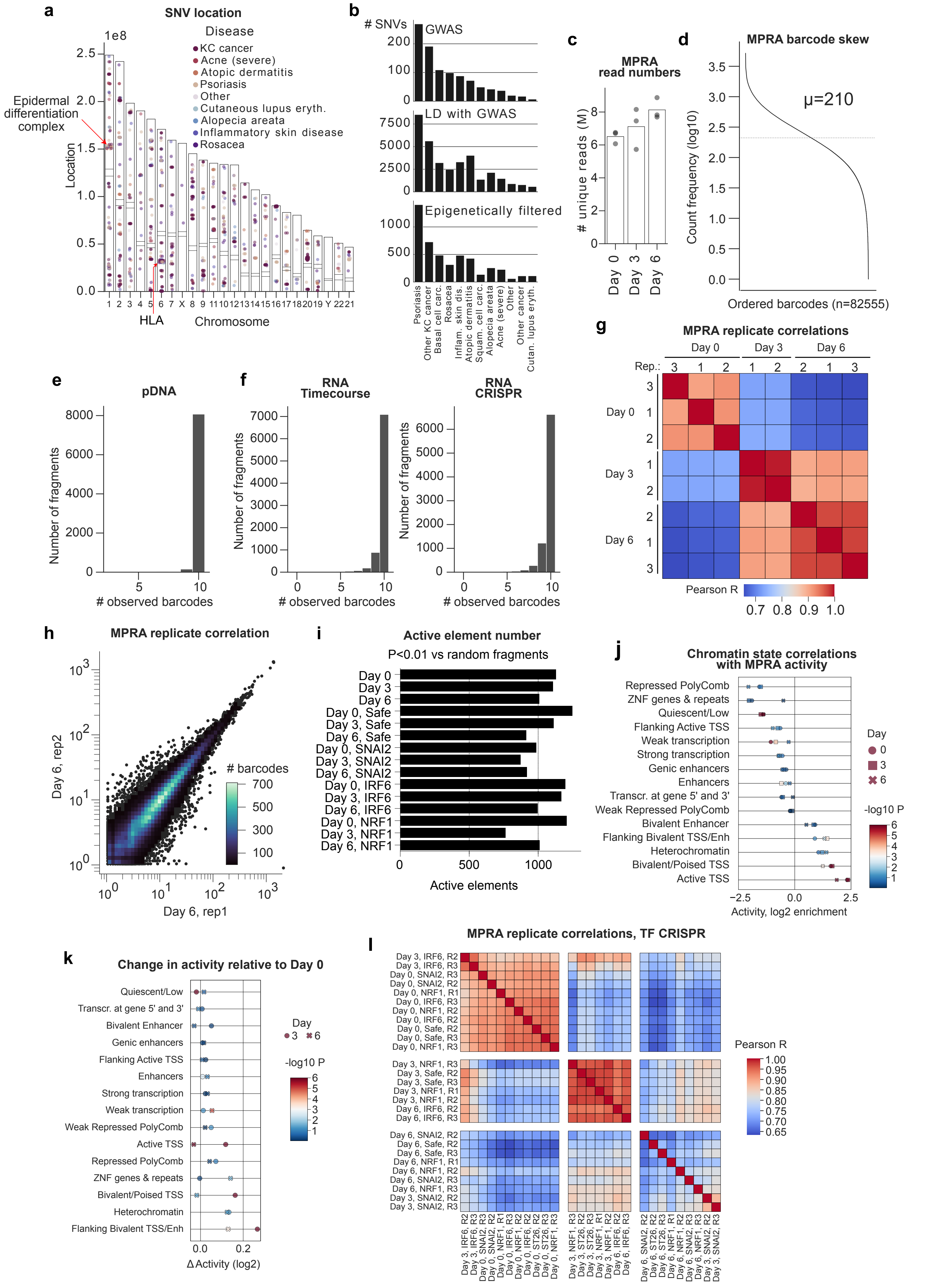

Figure S5

rs4687102 is a differential SNV linked to psoriasis and TP63

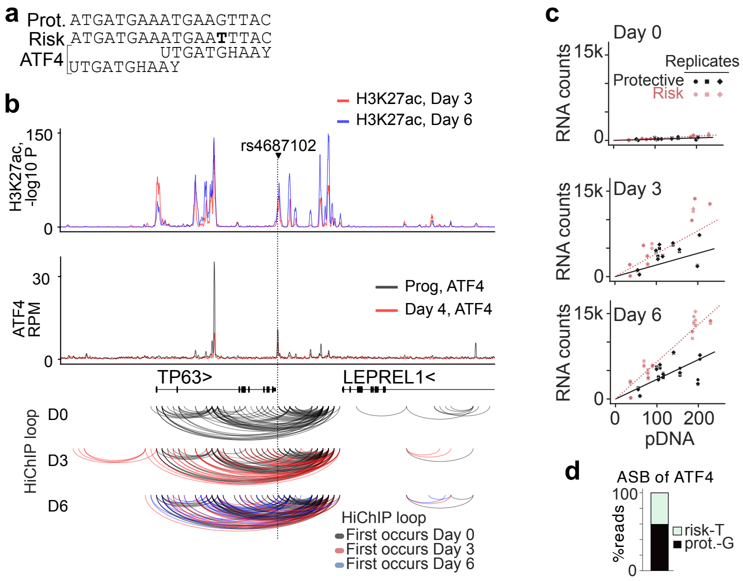

Figure S6

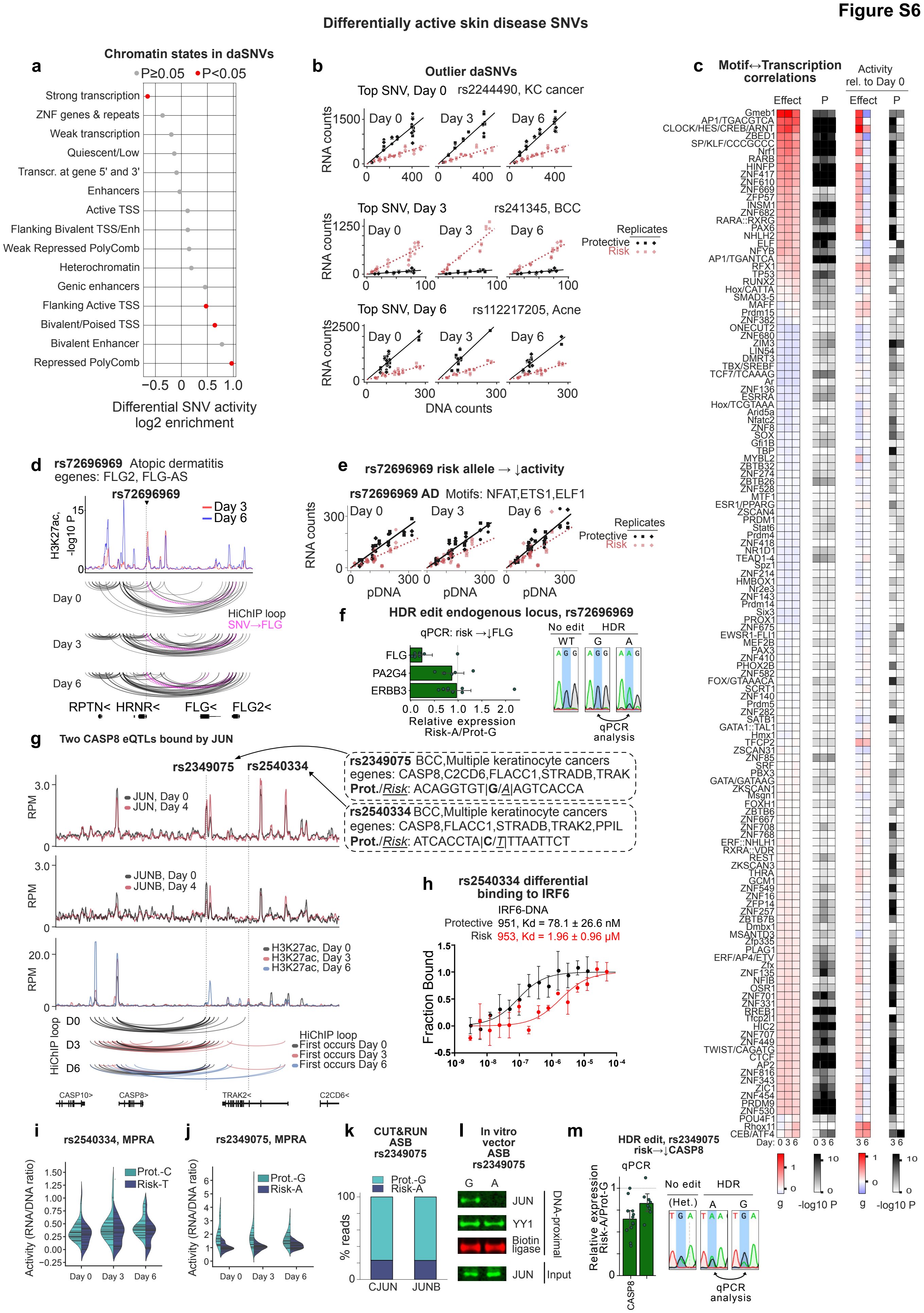

Differentially active skin disease SNVs
a
Chromatin states in daSNVs
P≥0.05 P<0.05
Strong transcription
ZNF genes & repeats
Weak transcription
Quiescent/Low
Transcr. at gene 5' and 3'
Enhancers
Active TSS
Flanking Bivalent TSS/Enh
Weak Repressed PolyComb
Heterochromatin
Genic enhancers
Flanking Active TSS
Bivalent/Poised TSS
Bivalent Enhancer
Repressed PolyComb
Differential SNV activity
log2 enrichment
b
Outlier daSNVs
Top SNV, Day 0 rs2244490, KC cancer
Top SNV, Day 3 rs241345, BCC
Top SNV, Day 6 rs112217205, Acne
RNA counts
DNA counts
Replicates
Protective
Risk
c
Motif↔Transcription correlations
Activity rel. to Day 0
Effect
P
d
rs72696969 Atopic dermatitis
egenes: FLG2, FLG-AS
H3K27ac, -log10 P
Day 3
Day 6
HiChIP loop
SNV→FLG
RPTN< HRNR< FLG< FLG2<
e
rs72696969 risk allele → ↓activity
rs72696969 AD Motifs: NFAT,ETS1,ELF1
pDNA
f
HDR edit endogenous locus, rs72696969
qPCR: risk →↓FLG
FLG
PA2G4
ERBB3
Relative expression
Risk-A/Prot-G
No edit HDR
WT G A
qPCR analysis
g
Two CASP8 eQTLs bound by JUN
rs2349075 rs2540334
JUN, Day 0
JUN, Day 4
JUNB, Day 0
JUNB, Day 4
H3K27ac, Day 0
H3K27ac, Day 3
H3K27ac, Day 6
RPM
HiChIP loop
First occurs Day 0
First occurs Day 3
First occurs Day 6
CASP10> CASP8> TRAK2< C2CD6<
rs2349075 BCC,Multiple keratinocyte cancers
egenes: CASP8,C2CD6,FLACC1,STRADB,TRAK
Prot./Risk: ACAGGTGT|G/A|AGTCACCA
rs2540334 BCC,Multiple keratinocyte cancers
egenes: CASP8,FLACC1,STRADB,TRAK2,PPIL
Prot./Risk: ATCACCTA|C/T|TTAATTCT
h
rs2540334 differential binding to IRF6
IRF6-DNA
Protective 951, Kd = 78.1 ± 26.6 nM
Risk 953, Kd = 1.96 ± 0.96 μM
Fraction Bound
i
rs2540334, MPRA
Prot.-C
Risk-T
Activity (RNA/DNA ratio)
j
rs2349075, MPRA
Prot.-G
Risk-A
k
CUT&RUN ASB rs2349075
% reads
CJUN JUNB
l
In vitro vector ASB rs2349075
G A
JUN
YY1
Biotin ligase
DNA-proximal
Input
m
HDR edit, rs2349075 risk→↓CASP8
qPCR
Relative expression Risk-A/Prot-G
CASP8
No edit (Het.)
HDR
qPCR analysis

Figure S7

CRISPR-FLOW of all transcription factors in keratinocytes

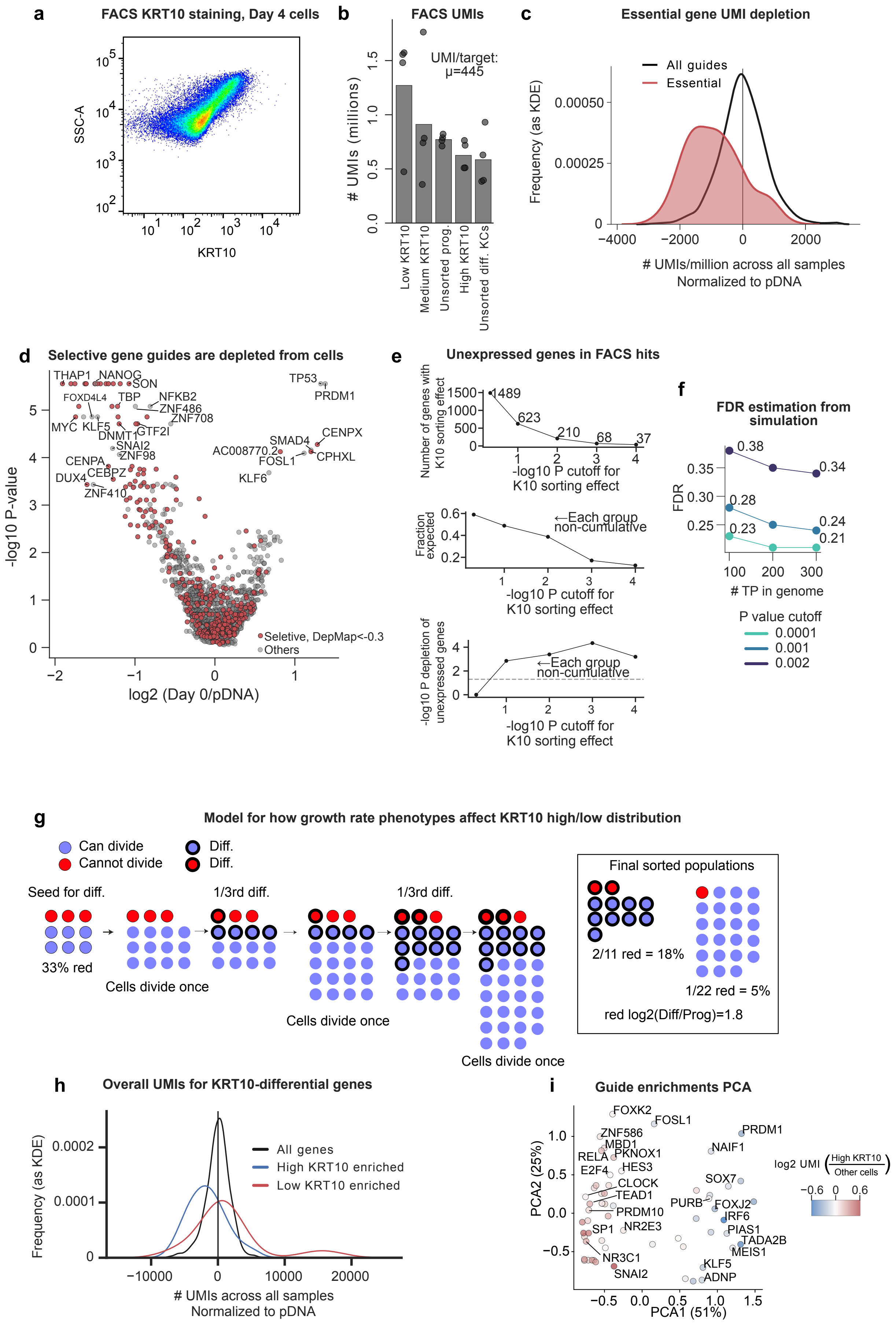

Figure S8

Overview of perturb-seq experiments

a

Differentiation	Technology	Library	# Usable cells	Cells/target (median)
Day 3	10X	402 TFs	79,577	187
Day 3	10X	100 TFs	16,447	144
Day 6	10X	402 TFs	26,574	62
Day 6	10X	100 TFs	12,582	112
Day 4	Split-pool	402 TFs	121,301	284
Day 4	Split-pool	100 TFs	18,398	165

b

RNA velocity analysis supports the pseudotime trajectory

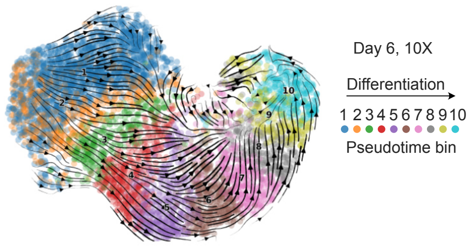

c

TF targets are successfully perturbed

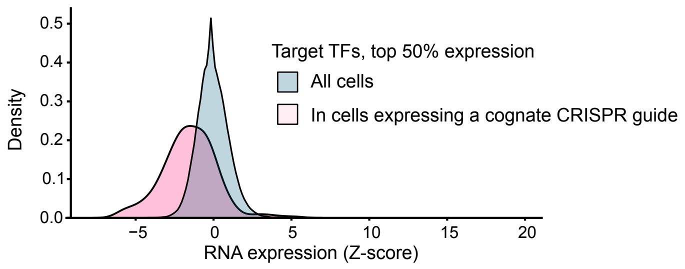

d

Day 3 ψ-effects by target TF

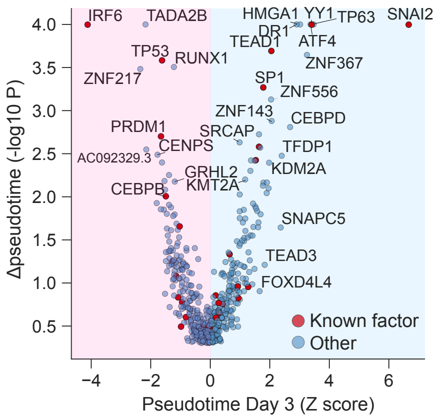

e

Day 6 ψ-effects by target TF

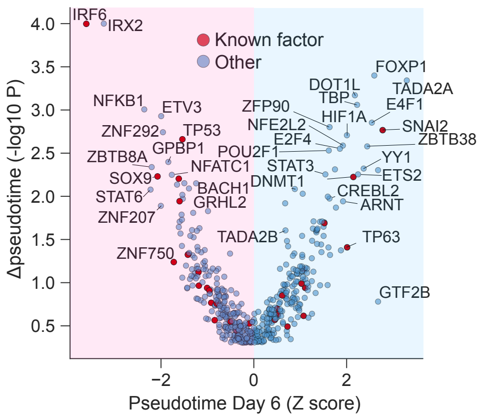

f

Droplet-based vs split-pool perturb-seq

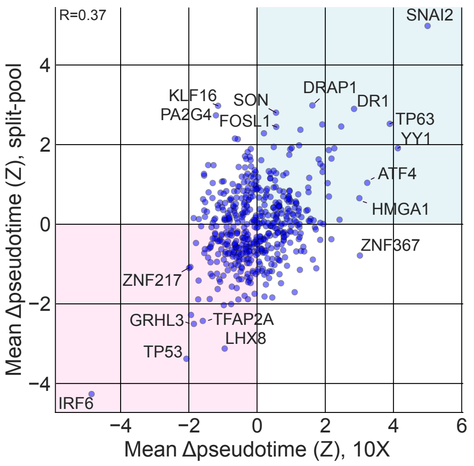

Figure S9

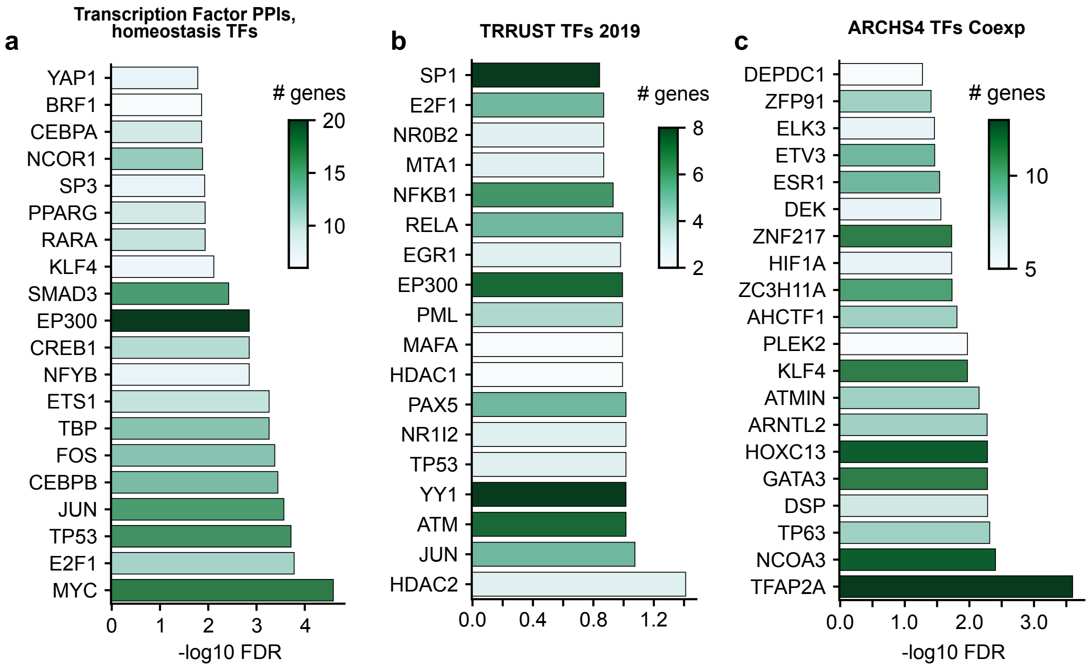
a
Transcription Factor PPIs, homeostasis TFs
YAP1
BRF1
CEBPA
NCOR1
SP3
PPARG
RARA
KLF4
SMAD3
EP300
CREB1
NFYB
ETS1
TBP
FOS
CEBPB
JUN
TP53
E2F1
MYC
0
1
2
3
4
-log10 FDR
genes
20
15
10
b
TRRUST TFs 2019
SP1
E2F1
NR0B2
MTA1
NFKB1
RELA
EGR1
EP300
PML
MAFA
HDAC1
PAX5
NR1I2
TP53
YY1
ATM
JUN
HDAC2
0.0
0.4
0.8
1.2
genes
8
6
4
2
c
ARCHS4 TFs Coexp
DEPDC1
ZFP91
ELK3
ETV3
ESR1
DEK
ZNF217
HIF1A
ZC3H11A
AHCTF1
PLEK2
KLF4
ATMIN
ARNTL2
HOXC13
GATA3
DSP
TP63
NCOA3
TFAP2A
0.0
1.0
2.0
3.0
-log10 FDR
genes
10
5

Figure S10

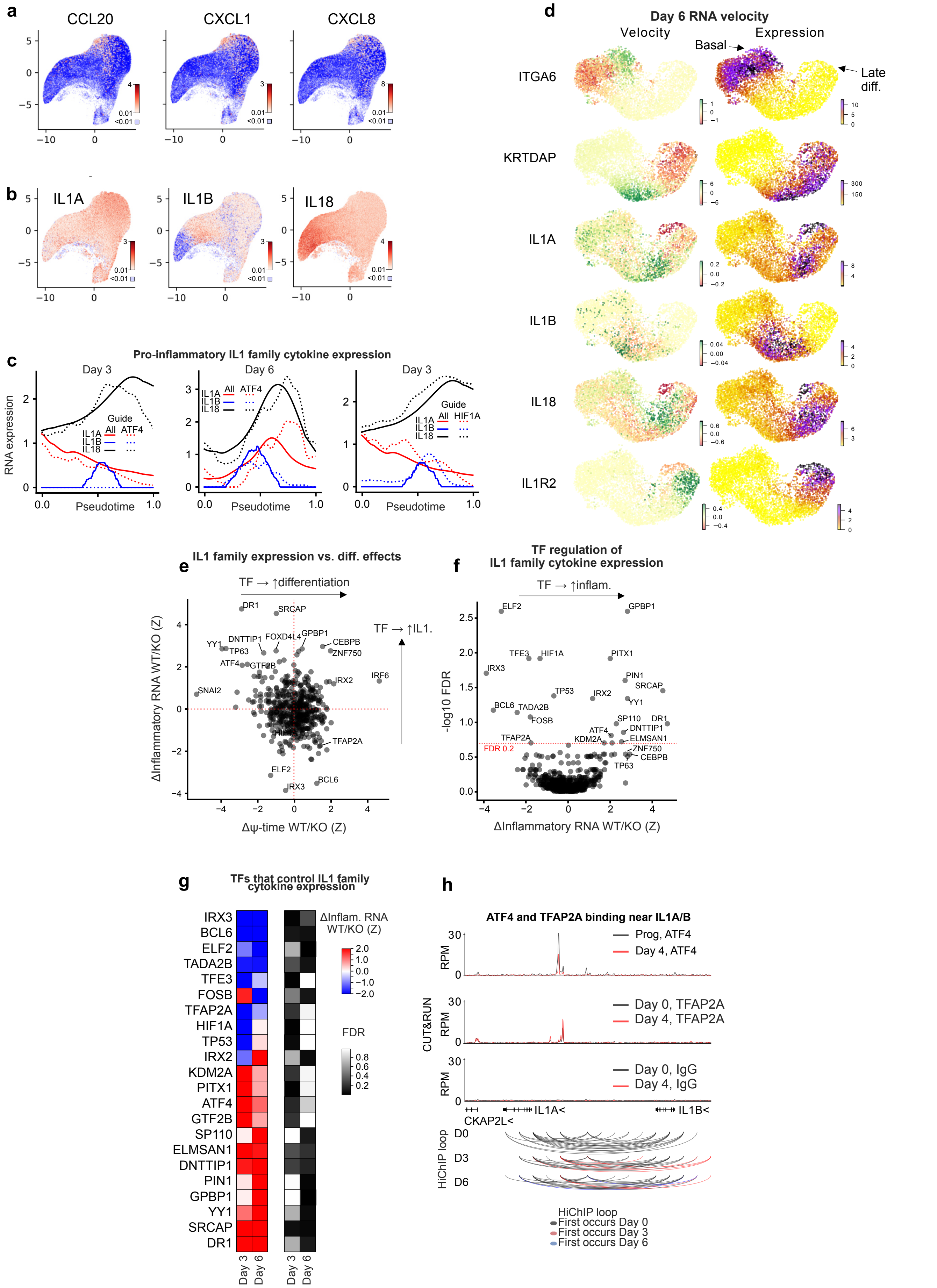

a
CCL20
CXCL1
CXCL8
b
IL1A
IL1B
IL18
c
Pro-inflammatory IL1 family cytokine expression
Day 3
Day 6
Day 3
RNA expression
Pseudotime
Guide
All ATF4
All HIF1A
IL1A
IL1B
IL18
d
Day 6 RNA velocity
Velocity
Expression
Basal
Late diff.
ITGA6
KRTDAP
IL1A
IL1B
IL18
IL1R2
e
IL1 family expression vs. diff. effects
TF → ↑differentiation
TF → ↑IL1.
ΔInflammatory RNA WT/KO (Z)
Δψ-time WT/KO (Z)
f
TF regulation of IL1 family cytokine expression
TF → ↑inflam.
-log10 FDR
FDR 0.2
ΔInflammatory RNA WT/KO (Z)
g
TFs that control IL1 family cytokine expression
IRX3
BCL6
ELF2
TADA2B
TFE3
FOSB
TFAP2A
HIF1A
TP53
IRX2
KDM2A
PITX1
ATF4
GTF2B
SP110
ELMSAN1
DNTTIP1
PIN1
GPBP1
YY1
SRCAP
DR1
ΔInflam. RNA WT/KO (Z)
FDR
Day 3
Day 6
h
ATF4 and TFAP2A binding near IL1A/B
CUT&RUN
RPM
Prog, ATF4
Day 4, ATF4
Day 0, TFAP2A
Day 4, TFAP2A
Day 0, IgG
Day 4, IgG
IL1A<
IL1B<
CKAP2L<
HiChIP loop
D0
D3
D6
HiChIP loop
First occurs Day 0
First occurs Day 3
First occurs Day 6

Novel skin TF functions and DNA binding

Figure S11

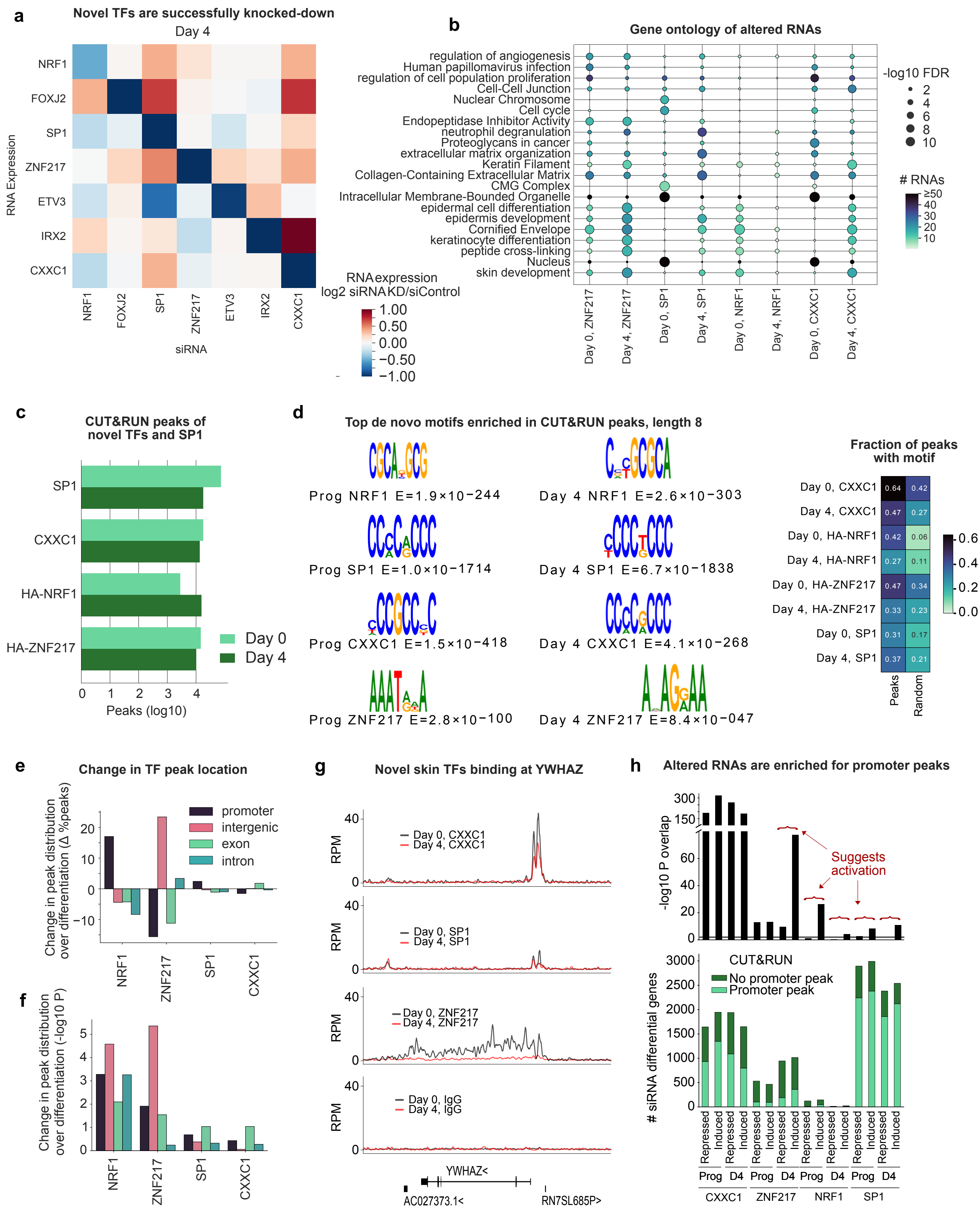

Figure S12

Homeostatic TF-DNA interactions across keratinocyte differentiation

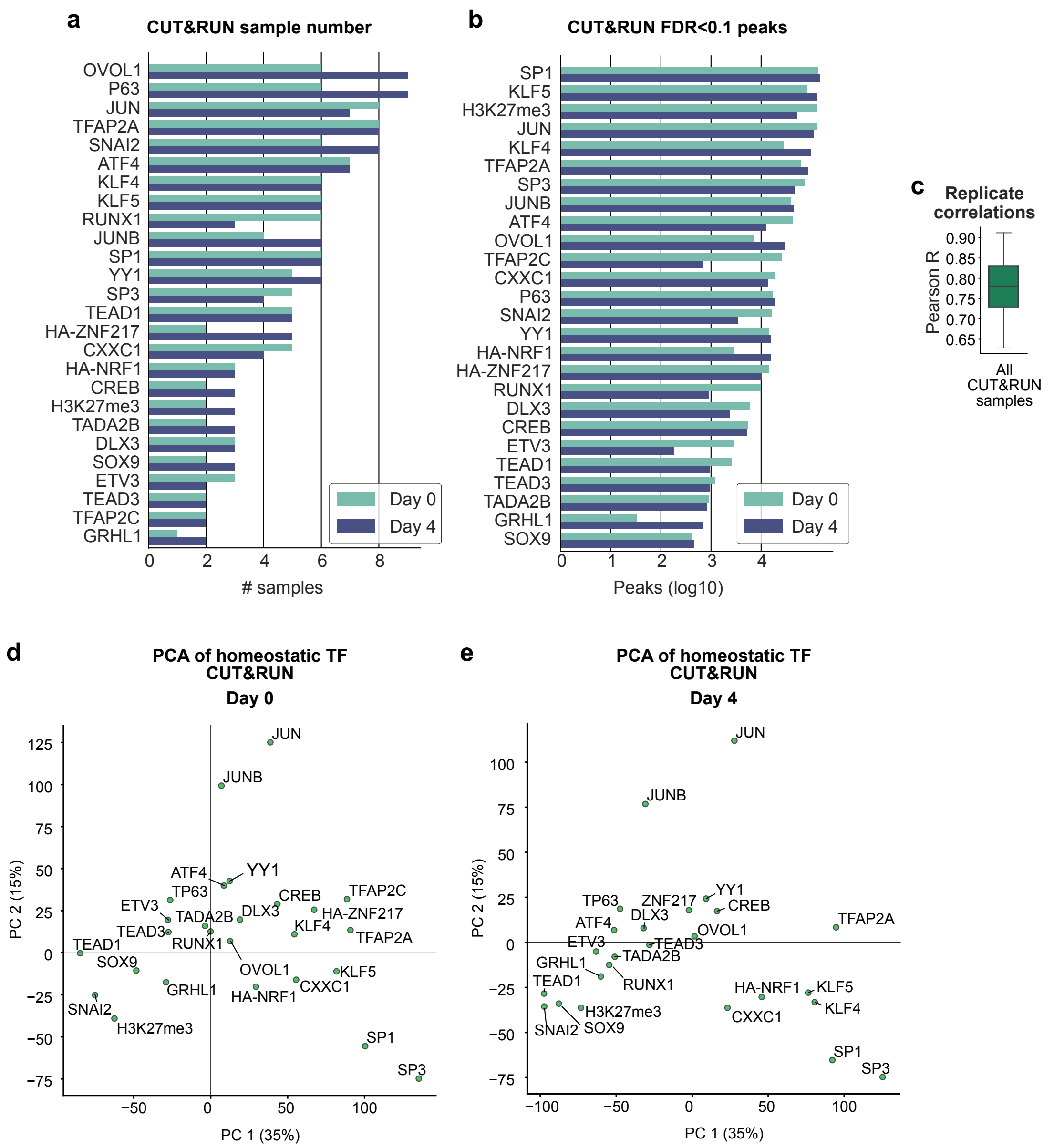

Figure S13

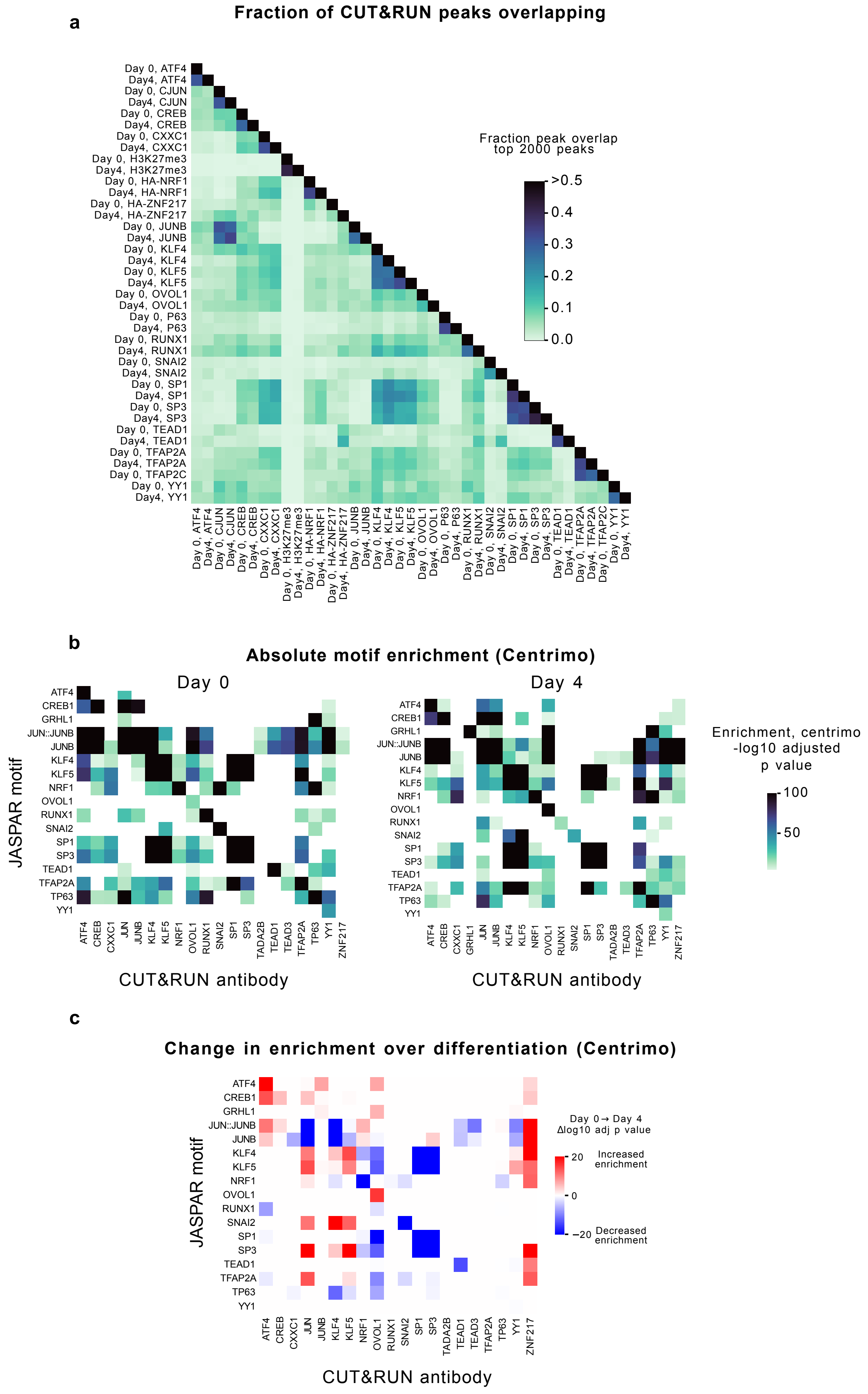
a
Fraction of CUT&RUN peaks overlapping
Day 0, ATF4
Day4, ATF4
Day 0, CJUN
Day4, CJUN
Day 0, CREB
Day4, CREB
Day 0, CXXC1
Day4, CXXC1
Day 0, H3K27me3
Day4, H3K27me3
Day 0, HA-NRF1
Day4, HA-NRF1
Day 0, HA-ZNF217
Day4, HA-ZNF217
Day 0, JUNB
Day4, JUNB
Day 0, KLF4
Day4, KLF4
Day 0, KLF5
Day4, KLF5
Day 0, OVOL1
Day4, OVOL1
Day 0, P63
Day4, P63
Day 0, RUNX1
Day4, RUNX1
Day 0, SNAI2
Day4, SNAI2
Day 0, SP1
Day4, SP1
Day 0, SP3
Day4, SP3
Day 0, TEAD1
Day4, TEAD1
Day 0, TFAP2A
Day4, TFAP2A
Day 0, TFAP2C
Day 0, YY1
Day4, YY1
Fraction peak overlap
top 2000 peaks
>0.5
0.4
0.3
0.2
0.1
0.0
b
Absolute motif enrichment (Centrimo)
Day 0
Day 4
JASPAR motif
ATF4
CREB1
GRHL1
JUN::JUNB
JUNB
KLF4
KLF5
NRF1
OVOL1
RUNX1
SNAI2
SP1
SP3
TEAD1
TFAP2A
TP63
YY1
ATF4
CREB
CXXC1
JUN
JUNB
KLF4
KLF5
NRF1
OVOL1
RUNX1
SNAI2
SP1
SP3
TADA2B
TEAD1
TEAD3
TFAP2A
TP63
YY1
ZNF217
CUT&RUN antibody
ATF4
CREB
CXXC1
GRHL1
JUN
JUNB
KLF4
KLF5
NRF1
OVOL1
RUNX1
SNAI2
SP1
SP3
TADA2B
TEAD3
TFAP2A
TP63
YY1
ZNF217
CUT&RUN antibody
Enrichment, centrimo
-log10 adjusted
p value
100
50
c
Change in enrichment over differentiation (Centrimo)
JASPAR motif
Day 0→ Day 4
Δlog10 adj p value
Increased enrichment
20
0
−20
Decreased enrichment
CUT&RUN antibody

Figure S14

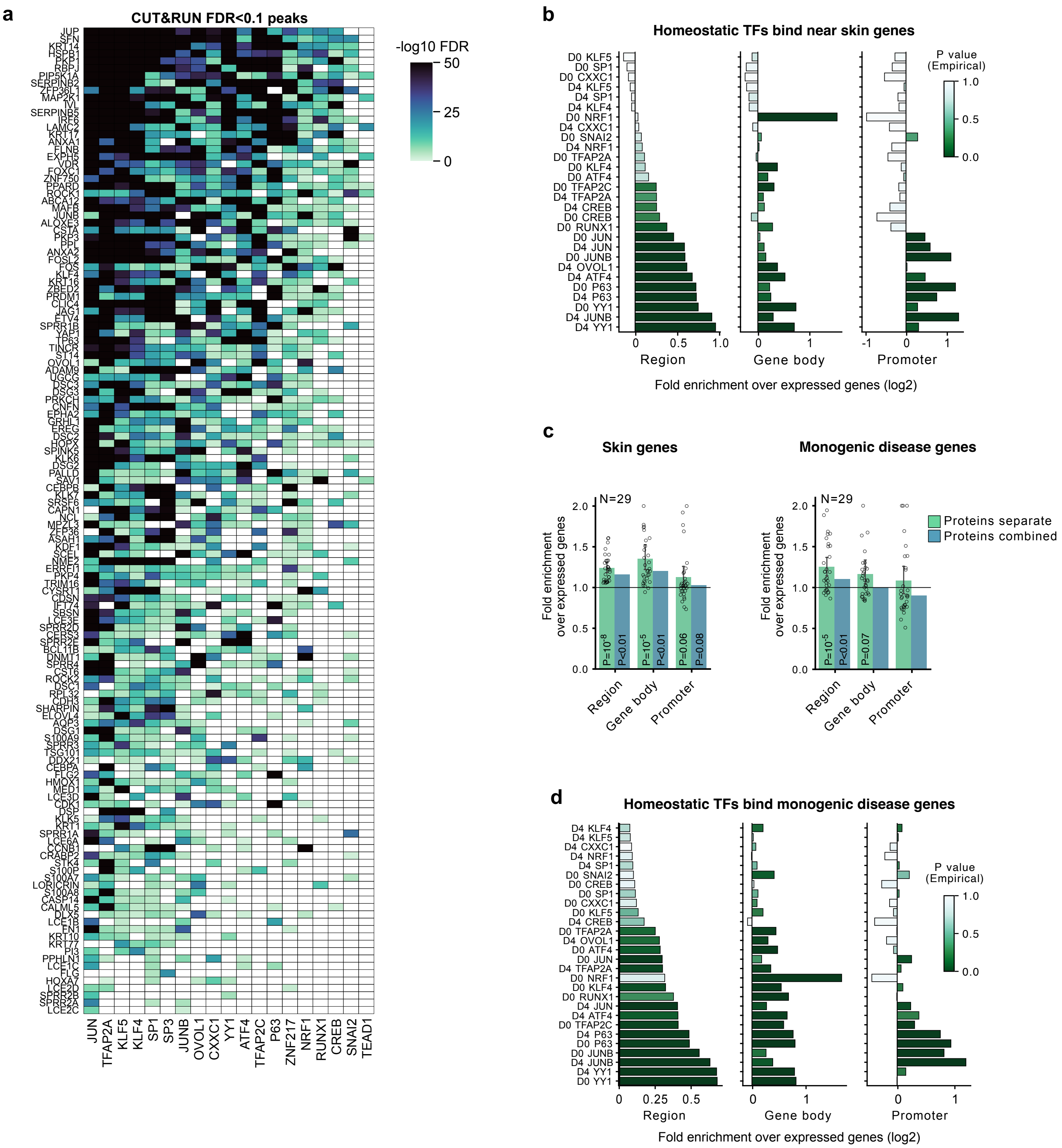

a
CUT&RUN FDR<0.1 peaks
-log10 FDR
50
25
0
JUN
TFAP2A
KLF5
KLF4
SP1
SP3
JUNB
OVOL1
CXXC1
YY1
ATF4
TFAP2C
P63
ZNF217
NRF1
RUNX1
CREB
SNAI2
TEAD1
b
Homeostatic TFs bind near skin genes
P value (Empirical)
1.0
0.5
0.0
D0 KLF5
D0 SP1
D0 CXXC1
D4 KLF5
D4 SP1
D4 KLF4
D0 NRF1
D4 CXXC1
D0 SNAI2
D4 NRF1
D0 TFAP2A
D0 KLF4
D0 ATF4
D0 TFAP2C
D4 TFAP2A
D4 CREB
D0 CREB
D0 RUNX1
D0 JUN
D4 JUN
D0 JUNB
D4 OVOL1
D4 ATF4
D0 P63
D4 P63
D0 YY1
D4 JUNB
D4 YY1
Region
Gene body
Promoter
Fold enrichment over expressed genes (log2)
c
Skin genes
Monogenic disease genes
N=29
Fold enrichment over expressed genes
Proteins separate
Proteins combined
P=10^-8
P<0.01
P=10^-5
P<0.01
P=0.06
P=0.08
P=10^-5
P<0.01
P=0.07
Region
Gene body
Promoter
d
Homeostatic TFs bind monogenic disease genes
P value (Empirical)
D4 KLF4
D4 KLF5
D4 CXXC1
D4 NRF1
D4 SP1
D0 SNAI2
D0 CREB
D0 SP1
D0 CXXC1
D0 KLF5
D4 CREB
D0 TFAP2A
D4 OVOL1
D0 ATF4
D0 JUN
D4 TFAP2A
D0 NRF1
D0 KLF4
D0 RUNX1
D4 JUN
D4 ATF4
D0 TFAP2C
D4 P63
D0 P63
D0 JUNB
D4 JUNB
D4 YY1
D0 YY1
Region
Gene body
Promoter
Fold enrichment over expressed genes (log2)

g

Figure S15

Promoter "buffering" of ASBs

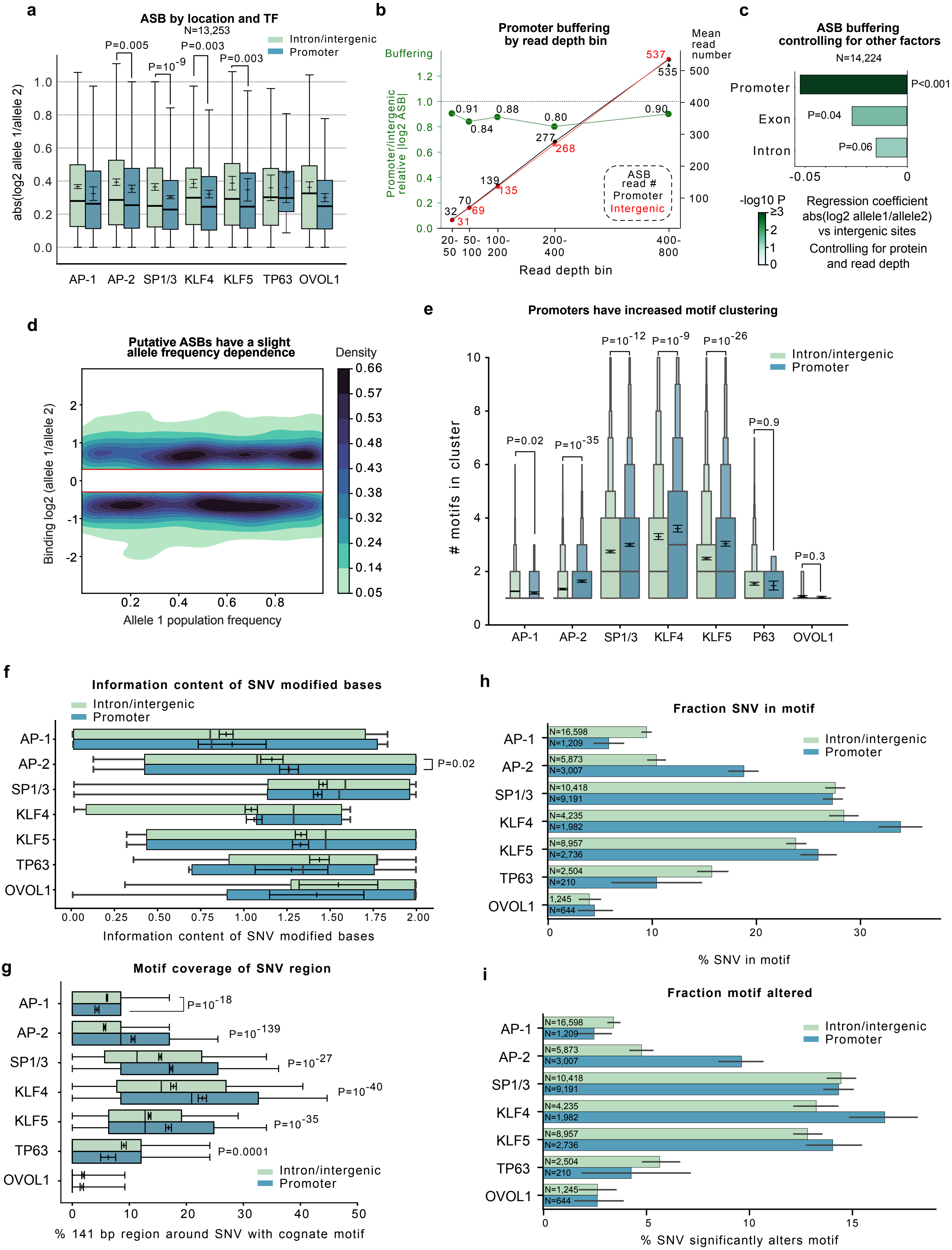

Figure S16

a

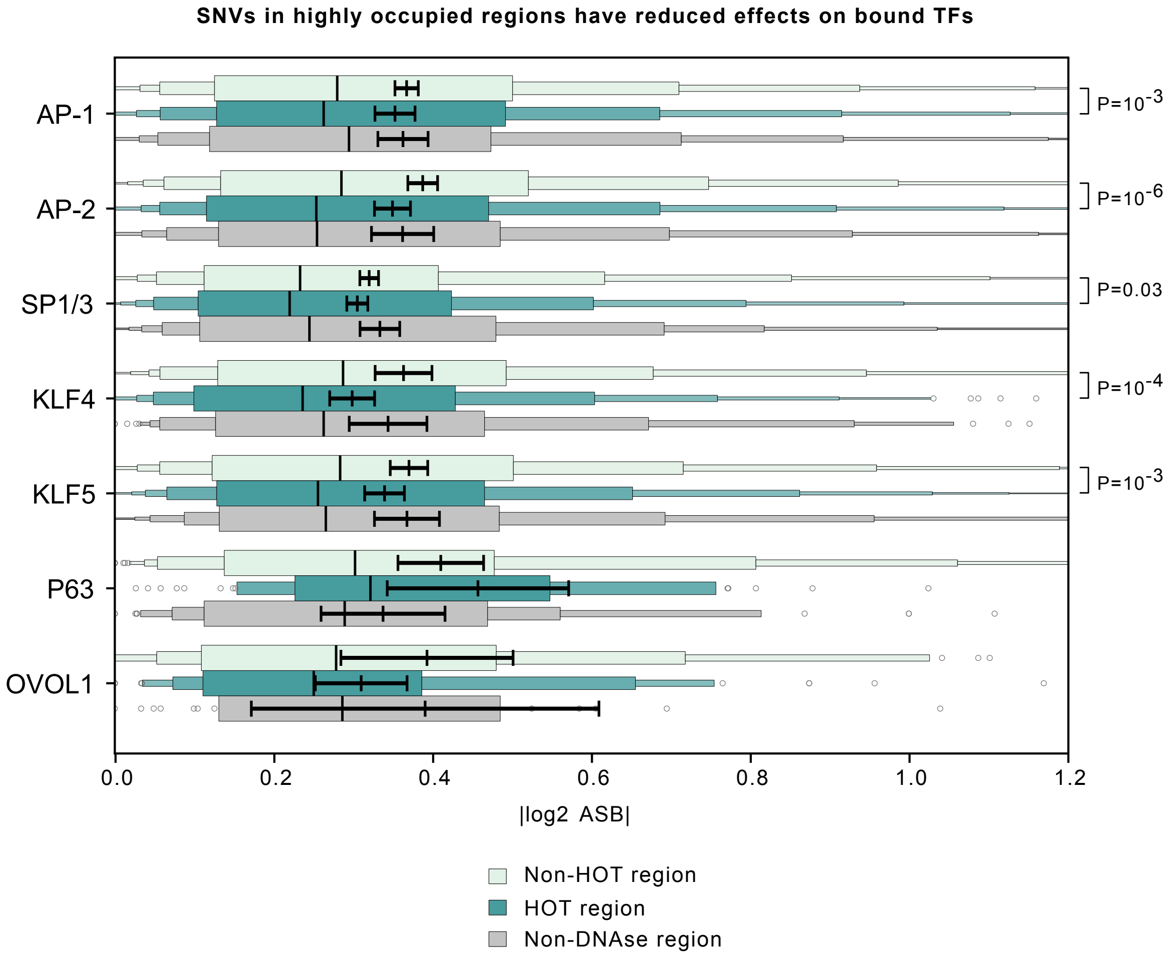

b

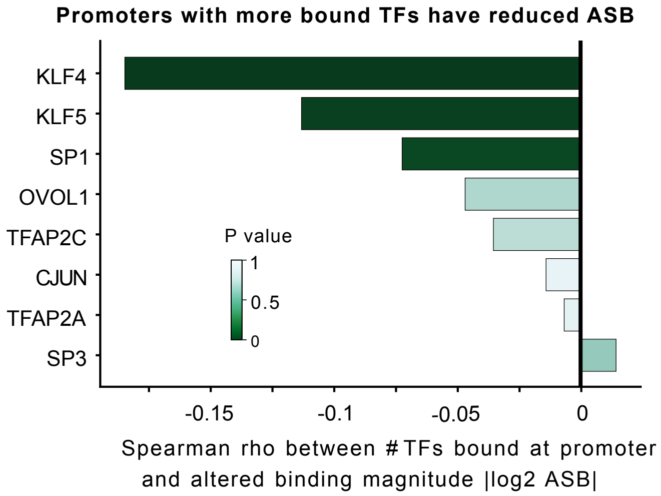

Figure S17

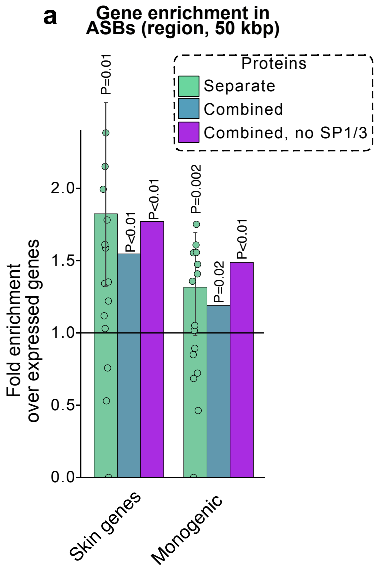

a
Gene enrichment in ASBs (region, 50 kbp)
Proteins
Separate
Combined
Combined, no SP1/3
P=0.01
P<0.01
P<0.01
P=0.002
P=0.02
P<0.01
Fold enrichment over expressed genes
2.0
1.5
1.0
0.5
0.0
Skin genes
Monogenic

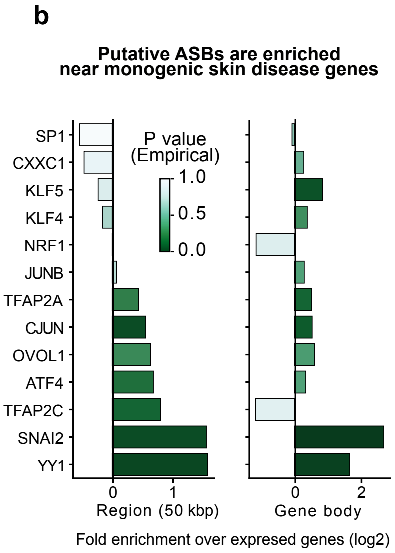

b
Putative ASBs are enriched near monogenic skin disease genes
SP1
CXXC1
KLF5
KLF4
NRF1
JUNB
TFAP2A
CJUN
OVOL1
ATF4
TFAP2C
SNAI2
YY1
P value (Empirical)
1.0
0.5
0.0
0
1
Region (50 kbp)
0
2
Gene body
Fold enrichment over expresed genes (log2)

Figure S18

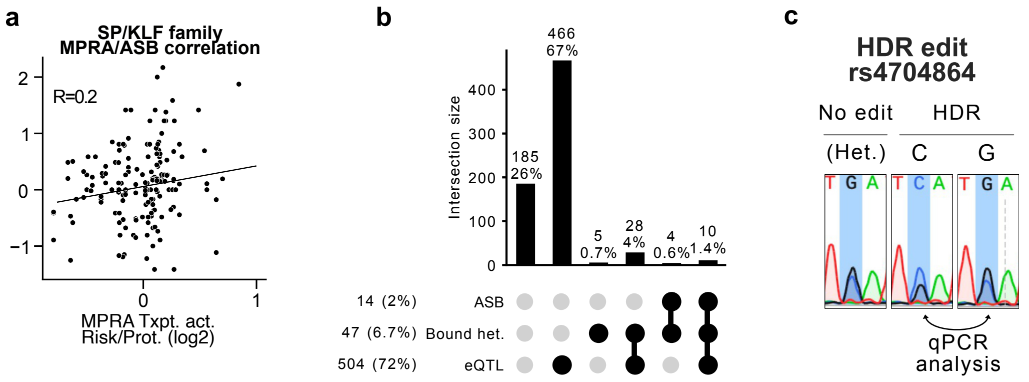
a
SP/KLF family
MPRA/ASB correlation
R=0.2
MPRA Txpt. act.
Risk/Prot. (log2)
b
Intersection size
466
67%
185
26%
5
0.7%
28
4%
4
0.6%
10
1.4%
14 (2%) ASB
47 (6.7%) Bound het.
504 (72%) eQTL
c
HDR edit
rs4704864
No edit
HDR
(Het.)
C
G
qPCR
analysis
