# Supplementary material for: Disease-linked regulatory DNA variants and homeostatic transcription factors in epidermis
Source: Nat Commun. 2025 Sep 25;16:8387. doi: 10.1038/s41467-025-63070-5 (PMC12462481; doi:10.1038/s41467-025-63070-5)
Supplement: Supplementary file 9 — Reporting Summary [file 41467_2025_63070_MOESM9_ESM.pdf]

nature portfolio

Corresponding author(s): Douglas F Porter, Paul A. Khavari

Last updated by author(s): Jul 21, 2025

Reporting Summary

Nature Portfolio wishes to improve the reproducibility of the work that we publish. This form provides structure for consistency and transparency in reporting. For further information on Nature Portfolio policies, see our Editorial Policies and the Editorial Policy Checklist.

Statistics

For all statistical analyses, confirm that the following items are present in the figure legend, table legend, main text, or Methods section.

n/a	Confirmed	
☐	☒	The exact sample size (*n*) for each experimental group/condition, given as a discrete number and unit of measurement
☐	☒	A statement on whether measurements were taken from distinct samples or whether the same sample was measured repeatedly
☐	☒	The statistical test(s) used AND whether they are one- or two-sided<br>*Only common tests should be described solely by name; describe more complex techniques in the Methods section.*
☐	☒	A description of all covariates tested
☐	☒	A description of any assumptions or corrections, such as tests of normality and adjustment for multiple comparisons
☐	☒	A full description of the statistical parameters including central tendency (e.g. means) or other basic estimates (e.g. regression coefficient) AND variation (e.g. standard deviation) or associated estimates of uncertainty (e.g. confidence intervals)
☐	☒	For null hypothesis testing, the test statistic (e.g. *F*, *t*, *r*) with confidence intervals, effect sizes, degrees of freedom and *P* value noted<br>*Give P values as exact values whenever suitable.*
☐	☒	For Bayesian analysis, information on the choice of priors and Markov chain Monte Carlo settings
☐	☒	For hierarchical and complex designs, identification of the appropriate level for tests and full reporting of outcomes
☐	☒	Estimates of effect sizes (e.g. Cohen's *d*, Pearson's *r*), indicating how they were calculated

Our web collection on statistics for biologists contains articles on many of the points above.

Software and code

Policy information about availability of computer code

Data collection	No software was used for data collection.
Data analysis	LDSC v.1.0.1, UMI-tools v. 1.1.5., Bowtie v. 1.3.1, MPRAnalyze v.1.9.1, BBMAP v.39.01, STAR v.2.7.3a, samtools v.1.18, Rsubread v.2.10.5, DESeq2 v.1.38.3, cutadapt v.4.5, bowtie2 v.2.4.2, MACS2 v.2.2.9.1, DREME v.5.5.4, AME v.5.5.3, BCFtools v.1.7, MEME v.5.1.1, rGREAT v.2.6.0.<br><br>Analysis scripts are available at https://github.com/khavarilab/cutandrun-analysis

For manuscripts utilizing custom algorithms or software that are central to the research but not yet described in published literature, software must be made available to editors and reviewers. We strongly encourage code deposition in a community repository (e.g. GitHub). See the Nature Portfolio guidelines for submitting code & software for further information.

Data

Policy information about availability of data

All manuscripts must include a data availability statement. This statement should provide the following information, where applicable:

- Accession codes, unique identifiers, or web links for publicly available datasets
- A description of any restrictions on data availability
- For clinical datasets or third party data, please ensure that the statement adheres to our policy

The CRISPR-flow guide library and MPRA sequencing data generated in this study has been deposited in the GEO database under accession code GSE255326

[https://www.ncbi.nlm.nih.gov/geo/query/acc.cgi?acc=GSE255326]. Under the same accession are additional processed data files for CUT&RUN and RNA-seq, including bigwig files of CUT&RUN data. Raw primary keratinocyte sequencing data is available under restricted access at accession code phs003977 [https://www.ncbi.nlm.nih.gov/projects/gap/cgi-bin/study.cgi?study_id=phs003977.v1.p1] to protect patient privacy; access can be obtained by request from dbGaP for general research use. MPRA data is available at the ARVID website [https://arvid-data.shinyapps.io/skin/] and reads-per-million normalized CUT&RUN data, along with peaks, can be accessed in the UCSC genome browser at at this link [https://genome.ucsc.edu/s/Max/cnr]. Allele specific binding events are included in Supplementary Data file 7. Source data are provided with this paper.

Research involving human participants, their data, or biological material

Policy information about studies with human participants or human data. See also policy information about sex, gender (identity/presentation), and sexual orientation and race, ethnicity and racism.

Reporting on sex and gender	The most typical source of primary keratinocytes is hospital circumcisions, which mostly limits us to male neonatal foreskin keratinocytes. However, there has not yet been a report of any major differences between male and female keratinocyte differentiation in vitro, suggesting our results are not restricted to one sex. In addition, samples were de-identified as per IRB protocols, so we do have not have sample-specific information on sex or gender.
Reporting on race, ethnicity, or other socially relevant groupings	No selection by race or ethnicity was performed and samples were de-identified as per IRB protocols, so we do not have sample-specific information.
Population characteristics	The source of primary keratinocytes was neonatal foreskin samples from discarded surgical samples from a California hospital in a diverse area.
Recruitment	Patients are asked by a physician if they would like to participate and there is no obvious self-selection bias.
Ethics oversight	The protocol was reviewed and approved by the Stanford Human Subjects Institutional Review Board (IRB), with approval number #35324.

Note that full information on the approval of the study protocol must also be provided in the manuscript.

Field-specific reporting

Please select the one below that is the best fit for your research. If you are not sure, read the appropriate sections before making your selection.

☒ Life sciences ☐ Behavioural & social sciences ☐ Ecological, evolutionary & environmental sciences

For a reference copy of the document with all sections, see nature.com/documents/nr-reporting-summary-flat.pdf

Life sciences study design

All studies must disclose on these points even when the disclosure is negative.

Sample size	No statistical procedure was used to determine sample size; sample sizes were as large as could be managed with the money and time available.
Data exclusions	No data was excluded, except that CUT&RUN samples were discarded if they did not separate from the negative controls by PCA plot and did not show enrichment of the cognate motif (if one existed).
Replication	Experiments were performed at least in duplicate, using multiple primary cell donors. In a few cases of CUT&RUN, there is only a single replicate for an antibody at a timepoint. When possible, the biosample was regressed out in DESeq2 analysis (design ~biosample + treatment vs ~biosample) to control for biological variation.
Randomization	No randomization was performed.
Blinding	In general, during library preparation, samples tubes were simply given numbers, and were only restored to their identity in the final analysis. The perturb-seq experiments cannot be blinded because there is only one sample type being processed. MPRA and CRISPR-flow experiments are similar in being a mostly a single library pool that is processed together. CUT&RUN experiments could be said to be effectively blinded because when antibody is added, it is done to a large number of PCR striptubes that are processed together and simply treated as numbers, with the note of what antibody being added being recorded for analysis later; the negative controls were often in random locations, and the library prep after digestion was often done later and possibly by another person, resulting in effective blinding for most of the preparation. Bulk RNA-seq samples were also prepared as numbered tubes and in a large batch, resulting in some blinding as to sample identity.

Reporting for specific materials, systems and methods

We require information from authors about some types of materials, experimental systems and methods used in many studies. Here, indicate whether each material, system or method listed is relevant to your study. If you are not sure if a list item applies to your research, read the appropriate section before selecting a response.

Materials & experimental systems

n/a	Involved in the study
☐	☒ Antibodies
☐	☒ Eukaryotic cell lines
☒	☐ Palaeontology and archaeology
☒	☐ Animals and other organisms
☒	☐ Clinical data
☒	☐ Dual use research of concern
☒	☐ Plants

Methods

n/a	Involved in the study
☒	☐ ChIP-seq
☒	☐ Flow cytometry
☒	☐ MRI-based neuroimaging

Antibodies

Antibodies used

All antibodies were used at 1µL in 50µL total volume (50X dilution) for CUT&RUN. anti-KRT10 was used at 1:100 dilution for CRISPR-flow staining.
anti-HA CST #3724
anti-H3K27me3 Active Motif #39055
anti-V5 CST #13202
anti-ATF4 ProteinTech 10835-1-AP
anti-SP1 ProteinTech 21962-1-AP
anti-CXXC1 ProteinTech 27963-1-AP
anti-SP3 ProteinTech 26584-1-AP
anti-KLF4 Sigma HPA002926
anti-KLF4 Bio-Techne AF3640
anti-c-JUN Abcam ab32137
anti-CREB1 Abcam ab32096
anti-YY1 Abcam ab109228
anti-SNAI2 CST #9585
anti-TFAP2A SCBT sc-12726/25343
anti-AHR SCBT sc-133088
anti-TEAD1 Abcam ab133533
anti-JUNB SCBT sc-8051
anti-OVOL1 ProteinTech 14082-1-AP
anti-TADA2B ProteinTech 67439-1-Ig
anti-SOX9 ProteinTech 17367-1-AP
anti-GRHL1 ProteinTech 17644-1-AP
anti-P63 CST #4892
anti-KLF5 ProteinTech 21017-1-AP
anti-RUNX1 ProteinTech 25315-1-AP
anti-TEAD3 CST 13224
anti-DLX3 ProteinTech 13261-3-AP
anti-ETV3 Aviva ARP85957
anti-KHDRBS1 Proteintech 10222-1-AP
anti-HOPX ProteinTech 11419-1-AP
IgG CST #2729
IgG SCBT sc-2025
anti-KRT10 Novus Bio NBP2-47825AF647

Validation

Antibodies were validated based on CUT&RUN quality metrics (e.g. cognate motif enrichment), or staining of differentiating keratinocytes (KRT10).

Eukaryotic cell lines

Policy information about cell lines and Sex and Gender in Research

Cell line source(s)

Lenti-X 293T from Takara Bio #632180 (female).

Human primary normal skin cells from Stanford University School of Medicine (male).

Authentication

Cell lines were not authenticated.

Mycoplasma contamination

Cells tested negative for mycoplasma.

Commonly misidentified lines
(See ICLAC register)

No commonly misidentified lines were used.

Plants

Seed stocks	*Report on the source of all seed stocks or other plant material used. If applicable, state the seed stock centre and catalogue number. If plant specimens were collected from the field, describe the collection location, date and sampling procedures.*
Novel plant genotypes	*Describe the methods by which all novel plant genotypes were produced. This includes those generated by transgenic approaches, gene editing, chemical/radiation-based mutagenesis and hybridization. For transgenic lines, describe the transformation method, the number of independent lines analyzed and the generation upon which experiments were performed. For gene-edited lines, describe the editor used, the endogenous sequence targeted for editing, the targeting guide RNA sequence (if applicable) and how the editor was applied.*
Authentication	*Describe any authentication procedures for each seed stock used or novel genotype generated. Describe any experiments used to assess the effect of a mutation and, where applicable, how potential secondary effects (e.g. second site T-DNA insertions, mosiacism, off-target gene editing) were examined.*
